# Apology on behalf of the publisher

**DOI:** 10.1080/03009734.2016.1207330

**Published:** 2016-07-07

In the previous issue of this journal (121-02), a number of errors were made.

- We like to make readers aware that Leif Jansson and Nils Welsh contributed as guest editors to the issue
- The title of the issue should have been *Remembering Claes Hellerström and those around him.* Unfortunately, this title was not placed on the front cover as it should have been
- In addition to this, a number of the ‘flashes’ were erroneously printed from the previous issue (121-01) on the front cover.

Taylor & Francis apologises for these errors.

